# Arterial spin labeling and diffusion-weighted imaging for identification of retropharyngeal lymph nodes in patients with nasopharyngeal carcinoma

**DOI:** 10.1186/s40644-022-00480-4

**Published:** 2022-08-17

**Authors:** Xiaoduo Yu, Fan Yang, Xue Liu, Yanfeng Zhao, Yujie Li, Meng Lin, Lizhi Xie, Yuqing Shang

**Affiliations:** 1 Department of Diagnostic Radiology, National Cancer Center/National Clinical Research Center for Cancer/Cancer Hospital, Chinese Academy of Medical Sciences and Peking Union Medical College, No17, Panjiayuannanli, Chaoyang district, Beijing, 100021 China; 2 MR Research China, GE Healthcare, No.1 Yongchang North Road, Beijing Economic-Technological Development Area, Beijing, 100176 China; 3 Department of Chronic Disease Epidemiology, Yale School of Public Health, Yale University, New Haven, CT CT06510 USA

**Keywords:** Nasopharyngeal neoplasms, Magnetic resonance imaging, Perfusion imaging, Diffusion magnetic resonance imaging

## Abstract

**Background:**

To evaluate the parameters derived from arterial spin labeling (ASL) and multi-b-value diffusion-weighted imaging (DWI) for differentiating retropharyngeal lymph nodes (RLNs) in patients with nasopharyngeal carcinoma (NPC).

**Methods:**

This prospective study included 50 newly diagnosed NPC and 23 healthy control (HC) participants. RLNs of NPC were diagnosed according to the follow-up MRI after radiotherapy. Parameters derived from ASL and multi-b-value DWI, and RLNs axial size on pre-treatment MRI among groups were compared. Receiver operating characteristic curve (ROC) was used to analyze the diagnostic efficiency.

**Results:**

A total of 133 RLNs were collected and divided into a metastatic group (n = 71) and two non-metastatic groups (n = 62, including 29 nodes from NPC and 33 nodes from HC). The axial size, blood flow (BF), and apparent diffusion coefficient (ADC) of RLNs were significantly different between the metastasis and the non-metastasis group. For NPC patients with a short axis < 5 mm or < 6 mm, or long axis < 7 mm, if BF > 54 mL/min/100 g or ADC ≤ 0.95 × 10^−3^ mm^2^/s, the RLNs were still considered metastatic. Compared with the index alone, a combination of size and functional parameters could improve the accuracy significantly, except the long axis combined with ADC; especially, combined size with BF exhibited better performance with an accuracy of 91.00–92.00%.

**Conclusions:**

ASL and multi-b-value DWI could help determine the N stage of NPC, while the BF combination with RLNs size may significantly improve the diagnostic efficiency.

**Supplementary Information:**

The online version contains supplementary material available at 10.1186/s40644-022-00480-4.

## Introduction

Nasopharyngeal carcinoma (NPC) is a type of head and neck cancer prevalent among the southern Chinese population and South-east Asians [1]. Due to high radio-sensitivity, NPC is mainly treated with radiotherapy, an effective treatment approach with promising outcomes [2, 3]. Therefore, imaging has a critical role in tumor staging, target region delineation, and therapeutic assessment [4, 5].

Patients with NPC often present with cervical lymphadenopathy (85–86.4% of cases); retropharyngeal lymph nodes (RLNs) and cervical level II nodes appear to be the first-echelon nodes [6–8]. RLNs metastases account for 63.6 to 75.1% of all cervical lymph node metastasis (LNM) among NPC patients [6, 7, 9] and are considered an independent prognostic factor for distant metastasis-free survival [8, 10]. Previous studies [7, 11] have suggested that LNM in NPC patients follows a predictable and orderly pattern and the rare occurrence of “skip” metastasis. Therefore, the presence of LNM in the first-echelon nodes could help the diagnosis of LNM in other regions of the neck. In addition, different treatment is used for patients with or without LNM. According to the NCCN guidelines [12], patients with stage I (T1N0M0) should receive definitive radiotherapy for the nasopharynx and elective radiotherapy for the neck, while those with advanced-stage should undergo chemotherapy based on radiotherapy. The needle biopsy on suspected cervical LNM contributes to the staging and improved prognosis in differentiated non-keratinizing squamous cell carcinoma on NPC [13]. However, unlike other palpable cervical lymph nodes in superficial areas, RLNs are difficult to approach with biopsy due to their anatomical location (usually located deep in the cervical soft tissue). Consequently, the diagnosis of RLNs depends on imaging, especially the MRI. Generally, RLNs are considered metastatic if any median RLNs are visible; lateral RLNs are diagnosed based on the size, nodal necrosis, and extracapsular spreading [9–11, 14–16]. Central necrosis has a 100% specificity for the diagnosis of LNM but a low incidence (19.0%) [15]. At present, there is still no standard criteria for the analysis of RLNs size with the short axis (5 mm or 6 mm) [9–11, 14–18].

Functional MRI can quantitatively probe the microenvironment of the tumor. Arterial spin label (ASL) utilizes radio frequency (RF) labeled arterial blood as an endogenous tracer to obtain the perfusion parameters blood flow (BF). In addition, 3D pseudo-continuous ASL (pCASL), based on fast spin-echo (FSE) series, have fewer artifacts suffered from the skull base and have higher repeatability, spatial resolution, and signal-to-noise ratio [19]. Multi-b-value diffusion-weighted imaging (DWI), which relies on mono-exponential and bi-exponential models, can be used to reflect the diffusion and perfusion of water molecules accurately.

This study aimed to compare metastatic and non-metastatic RLNs using parameters derived from ASL and multi-b-value DWI. We also evaluated the diagnostic efficiency of these two non-invasive imaging techniques on the differentiation of RLNs and additional help for the traditional size criterion.

## Materials and methods

### Patients

Our institutional review board approved this prospective study. Informed consent was obtained from all the participants before the MRI examination. A total of 57 consecutive patients with NPC, as proved by nasopharyngeal biopsy and pathology, were recruited from May 2015 to December 2016. Inclusion criteria were the following: (1) patients who underwent pre-treatment MRI, including the ASL and multi-b-value DWI; (2) patients treated by radiotherapy with or without chemotherapy; (3) patients who underwent an enhanced first follow-up MRI scan (3 months after the radiotherapy). To avoid interference of radiotherapy response of RLNs and surrounding tissue, we used both axial T2WI with fat suppression and enhanced series of follow-up MRI. Exclusion criteria were: (1) RLNs with short axis < 3 mm (n = 2) in case of any disruption on the measurement reliability of ASL and multi-b-value DWI; (2) lymph node inseparable from the primary nasopharyngeal tumor (n = 3). (3) if the RLNs showed stability in size on MR images 3 months after the radiotherapy but lost subsequent follow-up within 1 year (n = 2, 6, and 9 months after radiotherapy, respectively).

Finally, 50 NPC patients (36 male and 14 female, with a median age of 47 yrs.; ranging from 11–75 yrs.) were included in the study. According to the nasopharyngeal biopsies, 28 cases were identified as the undifferentiated type of non-keratinizing carcinoma (56.00%), 20 cases as the differentiated type of non-keratinizing carcinoma (40.00%), 1 case as the keratinizing carcinoma (0.02%), and 1 case without a specific type of tumor due to small biopsy tissue (0.02%). According to the 8th edition of the AJCC staging system [20], the tumor stage was summarized in Table 1. In addition, 25 HC participants without a history of nasopharyngeal diseases were recruited. All HC participants showed no obvious abnormality in nasopharynx before the MRI examination, and no nasopharyngeal lesion was found on at least 1 year of follow-up MRI. Two cases were excluded because there was no RLN with short axis > 3 mm. Therefore, 23 HC participants (11 male and 12 female) were included (with a median age of 38 yrs.; ranging from 26–65 yrs).

**Table 1:** TNM and clinical stage of all cases

T stage	T1	T2	T3	T4	
	11 (22.00%)	9 (18.00%)	13 (26.00%)	17 (34.00%)	
N stage	N0	N1	N2	N3a	
	0	24 (48.00%)	21 (42.00%)	5 (10.00%)	
M stage	M0	M1			
	52 (100%)	0			
Clinical stage	Stage I	Stage II	Stage III	Stage IVa	Stage IVb
	0	10 (20.00%)	19 (38.00%)	21 (42.00%)	0

### MR acquisitions

All image acquisitions were performed on a 3.0 T whole-body MR system (Discovery MR 750, GE Healthcare, Milwaukee, WI, USA) with an 8-channel head and neck phased array coil. Routine series were performed, including: (a) axial fast spin-echo (FSE) sequence T1WI; (b) FSE T1WI sagittal acquisition; (c) conventional axial fast recovery fast spin-echo (FRFSE) T2WI with iterative Dixon water-fat separation with echo asymmetry and least-squares estimation (IDEAL) (TR/TE = 4000 ms/85 ms, slice thickness/slice gap = 5 mm/1 mm) with the scan ranging from the bottom of frontal sinus to the oral pharynx (hereafter the conventional T2WI); (d) multi-b-value DWI was based on SE-EPI with the following parameters: TR/TE = 3500 ms/70 ms, slice thickness / slice gap = 5 mm/1 mm, field of view (FOV) = 26 cm, bandwidth = 250 Hz, matrix = 128 × 128, parallel imaging factor of 2, TA = 6 min; and 13 b values (NEX): 0 (1), 10 (2), 25 (2), 50 (2), 75 (2), 100 (1), 150 (1), 200 (1), 400 (1), 800 (4), 1000 (6), 1200 (6), and 1500 (6) s/mm^2^. The spatial coverage was the same as in the axial T2WI/IDEAL sequence, so the conventional T2WI was used as the reference to multi-b-value DWI during the data process.

- (e) The pseudo-continuous ASL sequence was performed with a 3D FSE spiral acquisition (axial; NEX = 3; bandwidth = 41.67 Hz; slice thickness/ slice gap = 3 mm/0 mm; ETL = 21; the number of slice = 30; FOV = 24 cm; image matrix = 288 × 192; TE = 11.1 ms; post-labeling delay = 1025 ms; TR/TA = 4326 ms/262 s). The scan ranged from the bottom of frontal sinus to oral pharynx. The labeling slab was automatically placed at 2.2 cm below the imaging plane.
- (f) Another axial T2WI/IDEAL series, as a reference to ASL for anatomic location, were performed with uniform scan range, thickness, and slice gap as those of ASL (hereafter the ASL matched T2WI).
- (g) Dynamic contrast-enhanced (DCE) MRI was only performed in newly diagnosed patients after the injection of Gadolinium-DTPA (Gd-DTPA).
- (e) A conventional axial enhanced scan was achieved with a 3D fast spoiled gradient-echo (FSPGR) (TR/TE = 320 ms/2.86 ms; slice thickness = 5 mm, with the same scan range as in the conventional T2WI, followed by the sagittal and coronal scan series. On follow-up MRI, after a manual injection of Gd-DTPA, axial, sagittal, and coronal T1WI/FSPGR series were scanned in turn, while DCE-MRI series was not included.

Parallel imaging using an array spatial sensitivity encoding technique (ASSET) was performed during the acquisition of the DWI and DCE-MRI series. The non-enhanced series was accomplished with the same parameters for all patients and HC participants, while the enhanced series was only conducted in patients.

### Criteria for RLNs of NPC patients

The RLNs diagnosis was assessed using the first follow-up MRI images, with referring to the contemporary response of the nasopharyngeal tumor. All patients achieved a complete response or partial response of nasopharyngeal neoplasm at 3 months after treatment, without stable disease or progressive disease, which was confirmed by nasopharyngoscopy. RLNs were considered metastatic if they significantly decreased in size (by ≥30%) on the first follow-up MRI, or exhibited stable size after radiotherapy but progressed during the subsequent follow-up MRI. In contrast, RLNs were considered non-metastatic if they had a stable size, and the patient remained disease-free during the subsequent follow-up [15, 21]. The axes in NPC patients and HC participants were measured and assessed (M.L., a radiologist with 21-year experience) according to the first follow-up MRI. The long axis was measured on conventional T2WI and the short axes were the widest diameter that was perpendicular to the long axis [15]. The same radiologist marked the location of RLNs on the two reference T2WI series of the pre-treatment MRI acquisitions.

### Data analysis

The RLNs on pre-treatment MR images were delineated by two independent observers (F.Y., 2-year experience; and X.Y., 18-year experience) according to the locations of RLNs marked by M.L.. The independent observers were blinded to the nature of the RLNs and the images and information of participants. All the measurements were performed using Functool software named as “ASL” and “MADC” on the vendor-supplied Advantage Workstation (ADW 4.6 version, GE, US).

Region of interest (ROI) was depicted as the entire region on the slice with the maximum area of RLNs to obtain the mean value of parameters. F.Y. first delineated the ROI of RLNs on ASL to acquire the mean BF value on the BF map derived by ASL. To prevent the memory interference, ROIs were depicted on RLNs on multi-b-value DWI to obtain four parameters (i.e., ADC [Apparent diffusion coefficient], D [Pure molecular diffusion], D* [Pseudo-diffusion coefficient], f [Perfusion fraction]) one month later. X.Y. used the same measurement method but different sequence order, i.e., the multi-b-value DWI sequence was used first, followed by the ASL sequence one month later. Besides the reference series of T2WI, other image contrasts such as T1WI were used to avoid obvious necrosis and artifacts, while enhanced images could not be served considering potential reviewer related bias.

### Statistical analysis

Statistical analyses were performed using SPSS Statistics Version 25.0, MedCalc software 15.8, and Graphpad prism5.0. Inter-observer consistency was investigated using a two-way random intra-class correlation (ICC). The averages of the measurements by two observers were used for further comparison. The two groups (metastasis and non-metastasis) were compared using an independent t-test (BF by ASL) and Mann-Whitney test (size and parameters by multi-b-value DWI) according to the conformation of normal distribution tested by Kolmogorov–Smirnov test. Comparisons among the three groups (metastasis and two non-metastasis groups) were verified by the Kruskal-Wallis test with post hoc Dunn’s explanatory analyses, together with Bonferroni correction. Delong’s test was used to compare the ROC among the significant indices. The diagnostic efficiency was calculated using RLNs size and functional parameters alone, and their combination. *P* values or adjusted *P* values (Kruskal-Wallis test) < 0.05 was considered as statistically significant.

## Results

### RLNs

A total of 133 lateral RLNs were analyzed. Seventy-one nodes (from 50 patients with stage N1 - N3) were metastatic with decreasing size (by ≥30%) on the first follow-up MRI. Twenty-nine nodes from 26 NPC patients were diagnosed as non-metastasis which exhibited stable size after radiotherapy and without progress during the subsequent follow-up MRI. In addition, there were 33 nodes from 23 HC participants.

All RLNs showed slight to moderate hyper-intensity on T2WI and mild to moderate enhancement on enhanced T1WI. Necrosis was observed within 20 metastatic lymph nodes. On the original ASL images, the signal intensity of the most metastatic RLNs was similar to the intensity found in primary tumors, and higher compared to those of surrounding muscle. At the same time, most of the non-metastatic RLNs had a similar or slightly higher signal intensity than those of the surrounding muscle. On multi-b-value DWI images, the signal intensity of all the RLNs was similar to those of the primary tumors and higher than those of surrounding muscles.

### Comparison of RLN size between groups

**Fig. 1:**
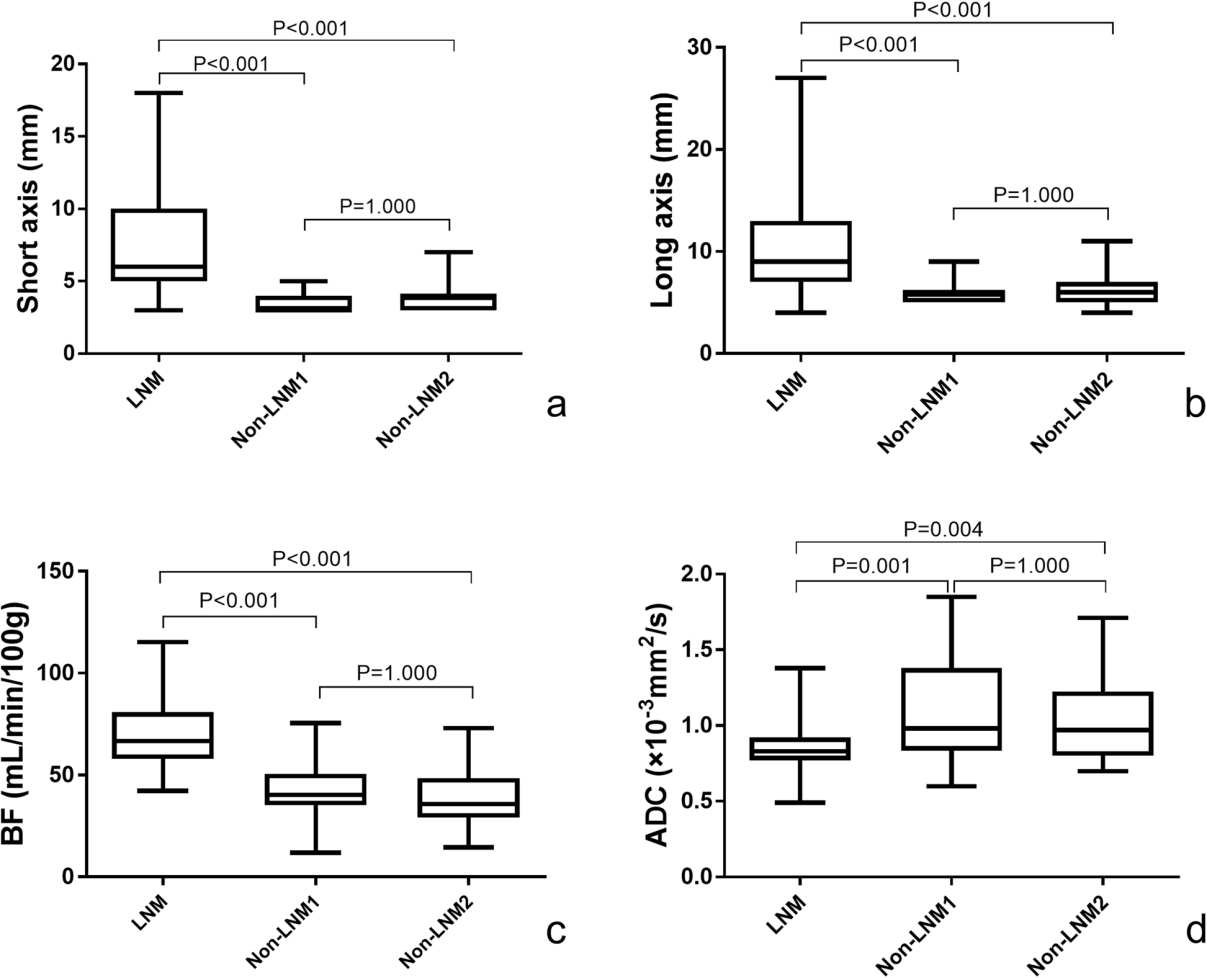
Box-plots of comparisons between three groups. Error bar plots showed the difference of short axis **(a)**, long axis **(b)**, mean BF **(c)**, and ADC **(d)** values of RLNs with LNM (metastatic RLNs from NPC patients), non-LNM1 (non-metastatic RLNs from NPC patients), and non-LNM2 (non-metastatic RLNs from healthy control participants)

The size of RLNs in NPC patients (before treatment) and HC participants is shown in Table S1, Fig. 1a**,** and Fig. 1b (An additional table shows this in more detail (see Additional file 1)). The size of RLNs was significantly different in the metastasis and non-metastasis group (*P* < 0.001 and = 0.001), but not between the two non-metastasis groups (*P* = 1.000). The data of RLNs size change and regression rate after radiotherapy and comparison among three groups are shown in Table S1 (An additional table shows this in more detail (see Additional file 1)). The number and percentage of metastatic RLNs in relation to the short and long axis are shown in Table 2. The percentage of metastatic RLNs with short axis <5 mm, <6 mm, and long axis <7 mm was 17/45, 27/56, and 8/32, respectively.

**Table 2:** RLNs diameter vs. the percentage of metastasis

	3 mm	4 mm	5 mm	6 mm	7 mm	8 mm	9 mm	≥10 mm
Short axis	1/18	16/27	10/11	13/13	2/2	6/6	5/5	18/18
Long axis	0	1/1	1/11	6/20	12/15	5/6	11/12	35/35

*RLNs* retropharyngeal lymph nodes

### RLNs parameters comparison between groups

BF from ASL and parameters from multi-b-value DWI before treatment is shown in Table S2, Fig. 1c, and Fig. 1d (An additional table shows this in more detail (see Additional file 1)). Good consistency (ICC: 0.885–0.979) was achieved between the two observers. For three groups comparison, BF and ADC were significantly different between the metastasis group and non-metastasis groups of patients or HCs, respectively (*P* < 0.001 or 0.004), and did not differ between the two non-metastasis groups. D, D*, and f did not show a statistical difference (Table 3, Table S2) (An additional table shows this in more detail (see Additional file 1)).

**Table 3:** LNM vs. Non-LNM

	Short axis (mm)	Long axis (mm)	BF (mL/min/100 g)	ADC (×10^−3^ mm^2^/s)	D (×10^−3^ mm^2^/s)	D^*^ (×10^−3^ mm^2^/s)	f
P	**<0.001**	**<0.001**	**<0.001**	**<0.001**	0.107	0.269	0.211
t/z	6.939	6.502	7.623^a^	−3.528	−1.610	−1.105	−1.25
Cutoff	≥5	≥7	>54	≤0.95			
AUC (95% CI)	0.937 (0.893–0.981)	0.913 (0.856–0.969)	0.910 (0.844–0.977)	0.727 (0.629–0.811)			

*ADC* apparent diffusion coefficient, *AUC* area under the curve, *BF* blood flow, *D* pure molecular diffusion; D* pseudo-diffusion coefficient, *f* perfusion fraction, *LNM* lymph node metastasis, *95% CI* 95% confidence interval

^a^Comparison was performed by an independent t-test

### Diagnostic efficiency

**Fig. 2:**
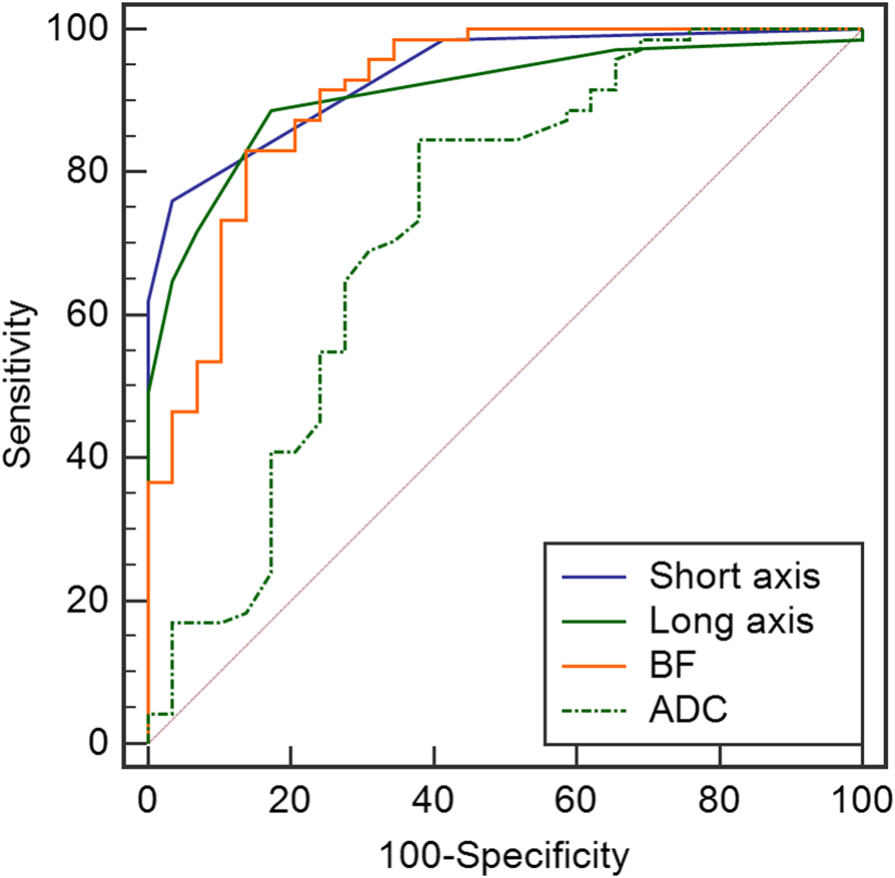
ROC by size, BF, and ADC to differentiate metastatic RLNs

Regarding NPC patients, metastatic RLNs had a larger size, higher BF, and lower ADC compared to non-metastatic RLNs (*P* < 0.001). Diagnostic criterion and efficiency is shown in Table 3, Table 4, and Fig. 2. The optimal cutoff of a single index to determine the metastasis according to short axis ≥ 5 mm, long axis ≥ 7 mm, BF > 54 mL/min/100 g, and ADC ≤ 0.95 × 10^−3^ mm^2^/s, respectively; i.e., setting short axis ≥ 6 mm resulted in a specificity of 100%. For ROC comparison, AUC by ADC was lower than the three parameters (short axis [*P* = 0.0005], long axis [*P* = 0.0051], and BF [*P* = 0.0036]) respectively, while no statistical difference was found among those three parameters.

**Table 4:** Diagnostic performance of parameters for identification of RLNs

Parameters (cutoff)	Sensitivity (*n* = 71)	Specificity (*n* = 29)	Accuracy (*n* = 100)
BF (>54 mL/min/100 g)	83.10 (59)	86.21 (25)	84.00 (84)
ADC (≤0.95 × 10^−3^ mm^2^/s)	84.51 (60)	62.07 (18)	78.00 (78)
Short axis (≥5 mm)	76.06 (54)	96.55 (28)	82.00 (82)
^a^Short axis + BF	95.77 (68)	82.76 (24)	92.00 (92)
^a^Short axis + ADC	98.59 (70)	62.07 (18)	88.00 (88)
Short axis (≥6 mm)	61.97 (44)	100 (29)	73.00 (73)
^a^Short axis + BF	94.31 (67)	86.21 (25)	92.00 (92)
^a^Short axis + ADC	97.18 (69)	62.07 (18)	87.00 (87)
Long axis (≥7 mm)	88.73 (63)	82.76 (24)	87.00 (87)
^a^Long axis + BF	100.00 (71)	68.97 (20)	91.00 (91)
^a^Long axis + ADC	95.77 (68)	51.72 (15)	83.00 (83)

*ADC* apparent diffusion coefficient, *BF* blood flow, *RLNs* retropharyngeal lymph nodes

^a^Combination criterion: for the size of RLNs less than their cutoff, if the functional parameters satisfied their cutoff, the RLNs were considered to metastatic; otherwise, they are non-metastatic

With a short axis <5 mm or <6 mm, or long axis <7 mm, BF >54 mL/min/100 g or ADC ≤0.95 × 10^−3^ mm^2^/s, the RLNs were still considered metastatic; otherwise, the RLNs were non-metastasis. Compared with the index alone, a combination of size and functional parameters improved the accuracy significantly, except the long axis combined with ADC. Mainly, combined size with BF exhibited better performance with significant improvement of accuracy of 91–92%; i.e., combining the long axis and BF provided the sensitivity of 100%.

**Fig. 3:**
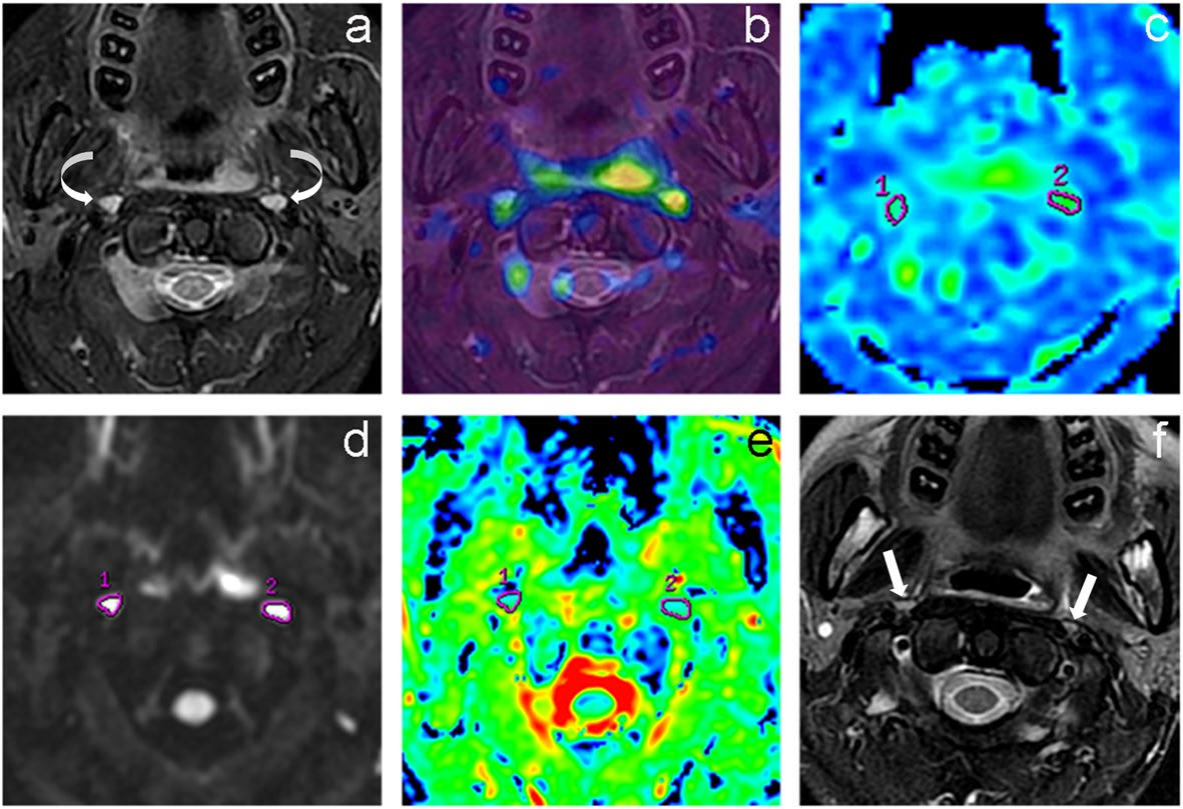
F29, NPC with bilateral metastatic RLNs. Axial T2WI before treatment **(a)** showed an irregular mass with heterogeneous slightly high signal intensity in the left lateral portion of the nasopharyngeal cavity and bilateral RLNs (curved arrows) with the size as long axis × short axis of 7 × 5 mm (right) and 8 × 5 mm (left), respectively. The primary tumor and RLNs demonstrated higher perfusion compared with the surrounding tissue on the fused image obtained by ASL and T2WI **(b),** and the map of BF **(c).** ROI of the RLNs were delineated on the map of BF (c) and multi-b-value DWI with b value at 800 s/mm^2^ **(d)**, acquiring at BF value of 60.63 mL/100 g/min, 67.71 mL/100 g/min, and ADC value of 0.808 × 10^−3^ mm^2^/s, 0.812 × 10^−3^ mm^2^/s on ADC map **(e**), respectively. Axial T2WI at three months after radiotherapy **(f)** showed the lymph nodes (stright arrows) were significantly reduced with the size of 4 × 2 mm (right) and 3 × 2 mm (left), respectively. Meanwhile, the tumor in the nasopharynx has complete regression

**Fig. 4:**
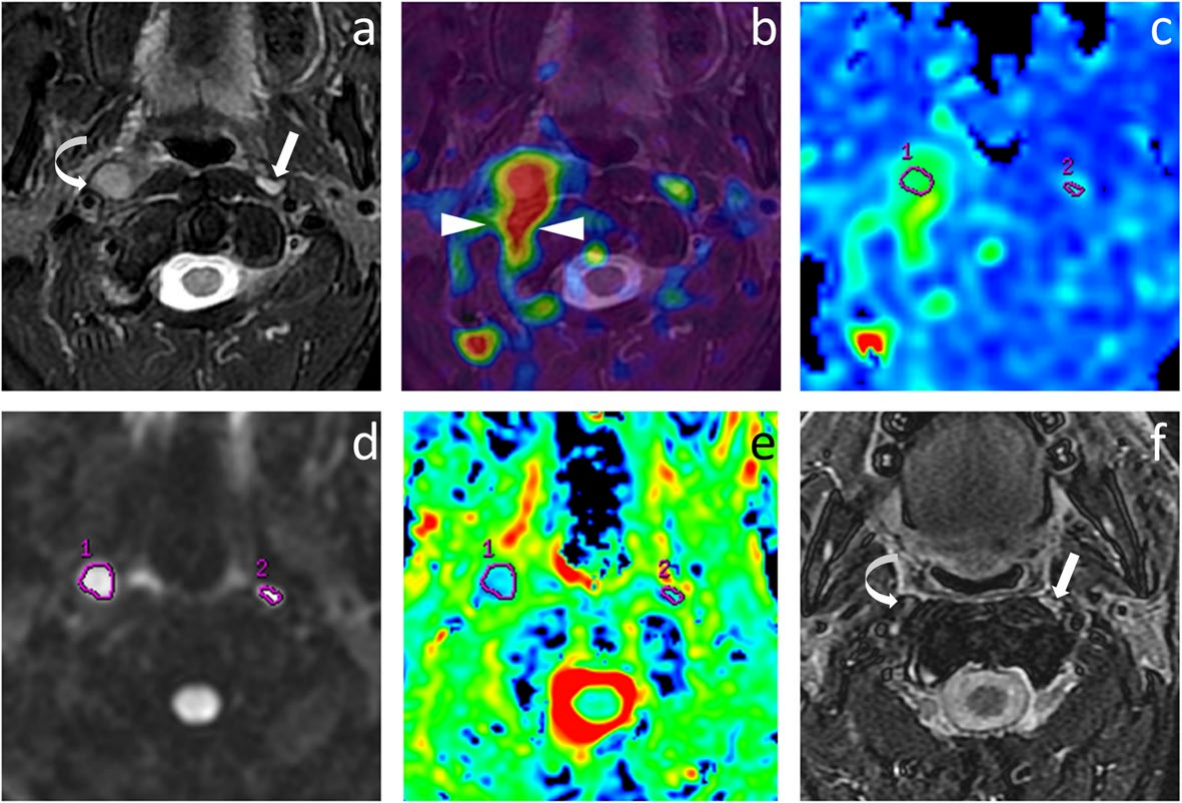
M50, NPC with right metastatic RLN, and left non-metastatic RLN. Axial T2WI before treatment **(a)** showed bilateral RLNs with slightly high heterogeneous signal intensity and size as long axis × short axis of 19 × 11 mm (right, curved arrow) and 7 × 5 mm (left, straight arrow), respectively. Both lymph nodes demonstrated higher perfusion than the surrounding tissue on the fused image by T2WI and ASL **(b),** and the map of BF **(c)**, especially the right one with remarkable pseudo-color contrast. On the fused image (b), the high perfusion area behind the right lymph node (arrowhead) was the internal carotid. ROI of the lymph nodes were delineated on the map of BF (c) and multi-b-value DWI with b value = 800 s/mm^2^ **(d)**, acquiring at BF value of 89.64 mL/100 g/min, 49.50 mL/100 g/min, and ADC value of 0.715 × 10^−3^ mm^2^/s, 0.893 × 10^−3^ mm^2^/s on ADC map **(e)**, respectively. Axial T2WI at three months after radiotherapy **(f)** showed the size was remarkably reduced to 6 × 3 mm on the right lymph node (curved arrow) and was reduced (but not significantly) to 7 × 4 mm on the left lymph node (straight arrow)

**Fig. 5:**
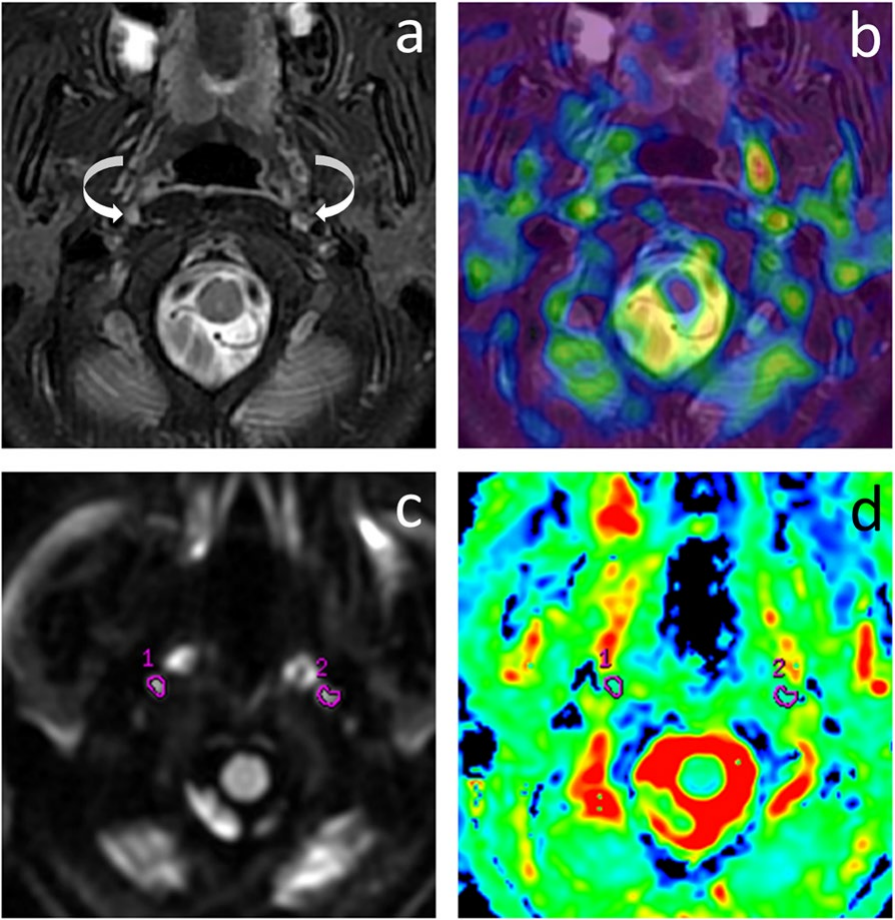
F28, healthy control with bilateral non-metastatic RLNs. Axial T2WI **(a)** shows bilateral RLNs (curved arrows) with heterogeneous slightly high signal intensity and size as long axis × short axis of 5 × 4 mm (right) and 7 × 5 mm (left), respectively. Both lymph nodes showed slightly higher perfusion compared with the surrounding tissue on the fused image by ASL and T2WI **(b)**, with a BF value of 42.5 mL/100 g/min and 32.14 mL/100 g/min. ROI of the lymph nodes was delineated on the multi-b-value DWI with b value = 800 s/mm^2^ **(c)**, acquiring at ADC value of 0.993 × 10^−3^ mm^2^/s and 0.874 × 10^−3^ mm^2^/s on ADC map **(d**), respectively

Representative image examples of metastatic and non-metastatic RLNs are shown in Figs. 3, 4 and 5.

## Discussion

The results of this study showed that metastatic RLNs had higher perfusion and lower diffusion compared to non-metastatic RLNs. When combining the size with functional information, the diagnostic efficiency in metastatic RLNs was significantly improved. Yet, no significance was found between the non-metastatic RLNs in patients and HC participants.

Previous studies [11, 15, 16, 22] suggested that the short axis of lymph node axis can be used as a size criterion to assess the RLNs metastases, revealing better accuracy than the long axis [15, 22]. However, there is no standard for the radiologic criterion. Lam *et al* suggested that 4 mm is the upper limit of normal RLN [14], while other studies suggested that metastatic RLNs can be defined as a short axis of 5 mm in NPC patients [9–11, 23]. In contrast, several studies revealed that short-axis ≥ 6 mm or 6.1 mm was the optimal size criteria to assess the RLNs metastases, with the accuracy of 87.5% [15] and 89.0%, or 89.1% [17], respectively, and sensitivity and specificity of 71 and 82% [18], respectively. Another study revealed the best performing cutoff was 4.5 mm for short axis and 6.5 mm for the long axis of RLNs in NPC patients [21]. Our study exhibited the optimal cutoff of the short axis, and the long axis of 5 mm and 7 mm resulted in an accuracy of 82 and 87%. In addition, all RLNs with a short axis ≥ 6 mm were metastatic. Our results also found that no statistical difference in ROC comparison between the short and long axis. Although this was evident for the long axis using cutoff at ≥7 mm with slightly higher accuracy than the short axis, the higher specificity (96.55 and 100%) was acquired by short axis when using ≥5 mm or ≥ 6 mm as a cutoff. Therefore, we suggested that both axes could be considered in clinical practice.

ASL can reliably reflect the tissue blood flow, having a good and consistent trend to DCE-MRI and MVD [24–27]. So far, few studies suggested that BF could be used to differentiate tumors of parotid gland [28] and nasal or sinonasal cavity (squamous cell carcinoma (SCC) and malignant lymphoma) [29], autoimmune thyroid disease (Graves’ disease VS. Hashimoto’s thyroiditis) [30], assess the changes perfusion between pre- and post-treatment in head and neck tumors, and to distinguish patients with residual tumors [31]. For NPC, studies proved that BF of ASL could help to delineate the tumor extent when fused T2WI with BF map [32], and improve the accuracy of stage assessment with moderate positive correlations to T stage and AJCC stage. Moreover, BF in tumor could also act as a noninvasive effective biomarker to predict tumor response to chemoradiotherapy in advanced-stage NPC [33].

By using perfusion CT, some studies revealed that BF value was more helpful in identifying metastatic lymph nodes compared to non-metastatic lymph nodes, such as axillary lymph nodes from breast cancer (161.60 mL/100 g/min and 76.18 mL/100 g/min) [34], cervical lymph nodes from head and neck SCC (HNSCC)(136.4 mL/100 g/min vs. 80.7 mL/100 g/min, and 114.62 mL/100 g/min vs. 67.82 mL/100 g/min) [35, 36]. Our ASL results were consistent with those results, yet our mean BF value was relatively low, which might be due to different equipment (MRI vs. CT), a contrast agent (RF-labeled arterial blood vs. iodinated contrast medium), and pharmacokinetic models (endogenous vs. exogenous).

The b-values is the key point for multi-b DWI using bi-exponential model to produce high-quality diffusion-weighted images and accurately quantify maps of the perfusion and diffusion parameters for ROI. However, so far, there is no clear consensus regarding the optimal choice of b value, even in the same disease on the previous studies [37]. In this study, a total of 13 b values were chosen including nine in the low range (0–400 s/mm2) in order to appropriately fit estimated perfusion-related parameters, and the high b value was set up to 1500 s/mm2 for reducing the artifacts of skull base. In our results, great inter-observer agreement was obtained with remarkably high ICC (0.885–0.979), which supported the reliability of the multi-b DWI data collected.

Despite the influence of microcirculation perfusion, ADC of DWI can reflect the diffusion movement of water molecules in tissues, thus supporting differentiation among cervical lymphadenopathy due to different causes [38–40]. A study on RLNs from NPC showed that the mean ADC value (< 0.929 × 10^−3^ mm^2^/s) contributes to the diagnosis of metastatic RLNs, with a very close cutoff of indices to our results [21]. Furthermore, their results demonstrated that ADC-based criteria would better predict RLN metastasis than size-based criteria.

The size and necrosis of lymph nodes may have a greater impact on the ADC value of metastatic lymph nodes. A small metastatic foci within a small metastatic lymph node may lead to an insignificant decrease of ADC value on the lymph node. Moreover, necrosis and liquefaction within lymph nodes decrease cell density and increase extracellular space, resulting in increased ADC value. Subsequently, some stratification studies based on the size and necrosis of the lymph nodes have been carried out. One study [41] revealed that the mean ADC value of metastatic lymph nodes of NPC was significantly lower compared to non-metastatic lymph nodes (0.878 × 10^−3^ mm^2^/s vs. 1.110 × 10^−3^ mm^2^/s) when lymph nodes had a shorter axis (from 5 to 10 mm) with AUC of 0.851. Another study [42] showed that the mean ADC value between metastatic and non-metastatic cervical lymph nodes with a short diameter (5 to 11 mm) and no necrosis was not different. In this study, ROIs were delineated to avoid the obvious necrosis, and the mean ADC values could be used as a useful index for the identification of RLNs.

Previously, Lai *et al* [43] have shown that multi-b-value DWI perfusion-related (f and D*) and quantitative DCE-MRI parameters (K^trans^, K_ep_, V_e_ and V_p_) were significantly correlated in the assessment of NPC, while D, f and D* could help with the prediction stage I/II from stage III/IV. Another study [44] has shown that the ADC, D, and D* values of NPC were significantly higher compared to nasopharyngeal lymphoma. There are still several studies arguing that perfusion-related parameters of multi-b-value DWI can help evaluate and predict radio-chemotherapy efficacy of NPC and metastatic lymph nodes [45–48]. Currently, no studies are using the bi-exponential model of multi-b-value DWI for the diagnosis of RLNs of NPC. There was a study on cervical lymph nodes from HNSCC [49] showing that ADC, D, and f were significantly lower in malignant lymph nodes than that in benign lymph nodes, whereas D* was significantly higher. However, our study showed D, D*, and f were not valuable criteria for identification.

A previous study showed that for RLNs with the short axis less than the cutoff of 6.1 mm, if the SUV mean ≥ 2.6, or long axis ≥ 8 mm and maximal coronal diameter ≥ 25 mm, the nodes were still positive, which resulted in improvement of accuracy compared with the short-axis alone (90.5% vs. 89.0%) [17]. Our data also showed that the combination of size (the short or long axis) with functional parameters could improve the accuracy for RLNs (except for combination of long axis with ADC), especially combined size with BF. i.e., RLNs with long axis of < 7 mm was non-metastatic if the BF < 54 mL/min/100 g. In our study, the optimal threshold of the short axis was ≥5 mm; however, whether setting diagnostic cutoff at 5 mm or 6 mm, a combination BF or ADC with a short axis can significantly improve the accuracy compared with the short-axis alone.

This study has some limitations. First, biopsy and histology were not applied due to the deep location of RLNs. Instead of using simple size diagnosis on pre-treatment imaging which has no consensus on cutoff, we adopted relatively objective criteria for metastatic RLNs based on the follow-up MRI. Considering no pathological confirmation, other interfering factors are inevibable. For example, nasopharyngeal inflammation may also cause RLNs enlargement and increased perfusion, which thus to be confused with metastasis. We have collected two non-metastasis group as control group from NPC patient and HC paticipants to avoid the confounding factors. Second, 5 mm slice thickness and 1 mm gap were set for DWI, considering the scan time and image quality. Thin slice thickness will increase the number of slice, thus to affect the accuracy of ROI delineation, especially in the skull base because of complex structure. Partial volume effects due to the slice thickness may have impact on the measurement of small RLNs, RLNs with short axis < 3 mm to reduce the influence. Third, the special cutoff (Table 4) may not be directly applied to other studies due to this preliminary exploration with relatively small cohort. The most significant point in our study was to explore the additional value of perfusion and diffusion information to RLNs size when the short or long axis of RLNs was less than the morphological criteria. Large sample and multi-center research are need to validate our results in the further study.

## Conclusion

The BF and ADC can reflect the difference of perfusion and diffusion between metastatic and non-metastatic RLNs. The implementation of ASL and DWI can be used as useful supplementary criteria for the conventional size to the N stage diagnosis and the treatment strategy, which can exhibit better performance in identifying RLNs with size less than the cutoff.

## Supplementary Information

**Additional file 1: Table S1.** RLNs size in the three groups. **Table S2.** Inter-observer consistency and comparisons of the parameters.

## Data Availability

Not applicable.

## Abbreviations

ASL: arterial spin labeling
DWI: diffusion-weighted imaging
RLNs: retropharyngeal lymph nodes
NPC: nasopharyngeal carcinoma
HC: healthy control
ROC: receiver operating characteristic
BF: blood flow
ADC: apparent diffusion coefficient
D: pure molecular diffusion
D*: pseudo-diffusion coefficient
f: perfusion fraction
LNM: lymph node metastasis
ROI: region of interest
AUC: area under the curve
CI: confidence interval

## References

[1] Jia WH, Huang QH, Liao J, Ye W, Shugart YY, Liu Q (2006). Trends in incidence and mortality of nasopharyngeal carcinoma over a 20-25 year period (1978/1983-2002) in Sihui and Cangwu counties in southern China. BMC Cancer.

[2] He J, Wu P, Tang Y, Liu S, Xie C, Luo S (2017). Chemoradiotherapy enhanced the efficacy of radiotherapy in nasopharyngeal carcinoma patients: a network meta-analysis. Oncotarget.

[3] Zhang B, Mo Z, Du W, Wang Y, Liu L, Wei Y (2015). Intensity-modulated radiation therapy versus 2D-RT or 3D-CRT for the treatment of nasopharyngeal carcinoma: A systematic review and meta-analysis. Oral Oncol.

[4] Yu E, O'Sullivan B, Kim J, Siu L, Bartlett E (2010). Magnetic resonance imaging of nasopharyngeal carcinoma. Expert Rev Anticancer Ther.

[5] Wei J, Pei S, Zhu X (2016). Comparison of 18F-FDG PET/CT, MRI and SPECT in the diagnosis of local residual/recurrent nasopharyngeal carcinoma: A meta-analysis. Oral Oncol.

[6] Wang X, Hu C, Ying H, He X, Zhu G, Kong L (2015). Patterns of lymph node metastasis from nasopharyngeal carcinoma based on the 2013 updated consensus guidelines for neck node levels. Radiother Oncol.

[7] Ho FC, Tham IW, Earnest A, Lee KM, Lu JJ (2012). Patterns of regional lymph node metastasis of nasopharyngeal carcinoma: a meta-analysis of clinical evidence. BMC Cancer.

[8] Chen B, Zhan Z, Pan J, Xiao Y, Tang L, Guo Q (2022). Re-evaluation of the prognostic significance of retropharyngeal node metastasis in nasopharyngeal carcinoma patients treated with intensity-modulated radiotherapy. Asia Pac J Clin Oncol.

[9] Liu LZ, Zhang GY, Xie CM, Liu XW, Cui CY, Li L (2006). Magnetic resonance imaging of retropharyngeal lymph node metastasis in nasopharyngeal carcinoma: patterns of spread. Int J Radiat Oncol Biol Phys.

[10] Tang L, Li L, Mao Y, Liu L, Liang S, Chen Y (2008). Retropharyngeal lymph node metastasis in nasopharyngeal carcinoma detected by magnetic resonance imaging : prognostic value and staging categories. Cancer.

[11] Tang L, Mao Y, Liu L, Liang S, Chen Y, Sun Y (2009). The volume to be irradiated during selective neck irradiation in nasopharyngeal carcinoma: analysis of the spread patterns in lymph nodes by magnetic resonance imaging. Cancer.

[12] Kallehauge J, Nielsen T, Haack S, Peters DA, Mohamed S, Fokdal L (2013). Voxelwise comparison of perfusion parameters estimated using dynamic contrast enhanced (DCE) computed tomography and DCE-magnetic resonance imaging in locally advanced cervical cancer. Acta Oncol.

[13] Lv JW, Zhou GQ, Chen YP, Tang LL, Mao YP, Chen L (2017). Refining the Role of Lymph Node Biopsy in Survival for Patients with Nasopharyngeal Carcinoma: Population-Based Study from the Surveillance Epidemiology and End-Results Registry. Ann Surg Oncol.

[14] Lam WW, Chan YL, Leung SF, Metreweli C (1997). Retropharyngeal lymphadenopathy in nasopharyngeal carcinoma. Head Neck.

[15] Zhang GY, Liu LZ, Wei WH, Deng YM, Li YZ, Liu XW (2010). Radiologic criteria of retropharyngeal lymph node metastasis in nasopharyngeal carcinoma treated with radiation therapy. Radiology.

[16] Ng SH, Chang JT, Chan SC, Ko SF, Wang HM, Liao CT (2004). Nodal metastases of nasopharyngeal carcinoma: patterns of disease on MRI and FDG PET. Eur J Nucl Med Mol Imaging.

[17] Wang YW, Wu CS, Zhang GY, Chang CH, Cheng KS, Yao WJ (2016). Can Parameters Other than Minimal Axial Diameter in MRI and PET/CT Further Improve Diagnostic Accuracy for Equivocal Retropharyngeal Lymph Nodes in Nasopharyngeal Carcinoma?. PLoS One.

[18] Chen J, Luo J, He X, Zhu C (2020). Evaluation of Contrast-Enhanced Computed Tomography (CT) and Magnetic Resonance Imaging (MRI) in the Detection of Retropharyngeal Lymph Node Metastases in Nasopharyngeal Carcinoma Patients. Cancer Manag Res.

[19] Wu B, Lou X, Wu X, Ma L (2014). Intra- and interscanner reliability and reproducibility of 3D whole-brain pseudo-continuous arterial spin-labeling MR perfusion at 3T. J Magn Reson Imaging.

[20] Pan JJ, Ng WT, Zong JF, Chan LL, O'Sullivan B, Lin SJ (2016). Proposal for the 8th edition of the AJCC/UICC staging system for nasopharyngeal cancer in the era of intensity-modulated radiotherapy. Cancer.

[21] Li H, Liu XW, Geng ZJ, Wang DL, Xie CM (2015). Diffusion-weighted imaging to differentiate metastatic from non-metastatic retropharyngeal lymph nodes in nasopharyngeal carcinoma. Dentomaxillofac Radiol.

[22] van den Brekel MW, Stel HV, Castelijns JA, Nauta JJ, van der Waal I, Valk J (1990). Cervical lymph node metastasis: assessment of radiologic criteria. Radiology.

[23] King AD, Ahuja AT, Leung SF, Lam WW, Teo P, Chan YL (2000). Neck node metastases from nasopharyngeal carcinoma: MR imaging of patterns of disease. Head Neck.

[24] Dangouloff-Ros V, Deroulers C, Foissac F, Badoual M, Shotar E, Grevent D (2016). Arterial Spin Labeling to Predict Brain Tumor Grading in Children: Correlations between Histopathologic Vascular Density and Perfusion MR Imaging. Radiology.

[25] Zhang Y, Kapur P, Yuan Q, Xi Y, Carvo I, Signoretti S (2016). Tumor Vascularity in Renal Masses: Correlation of Arterial Spin-Labeled and Dynamic Contrast-Enhanced Magnetic Resonance Imaging Assessments. Clin Genitourin Cancer.

[26] Lin M, Yu X, Luo D, Ouyang H, Xie L, Wu B (2018). Investigating the correlation of arterial spin labeling and dynamic contrast enhanced perfusion in primary tumor of nasopharyngeal carcinoma. Eur J Radiol.

[27] Xiao B, Wang P, Zhao Y, Liu Y, Ye Z (2020). Nasopharyngeal carcinoma perfusion MRI: Comparison of arterial spin labeling and dynamic contrast-enhanced MRI. Medicine (Baltimore).

[28] Kato H, Kanematsu M, Watanabe H, Kajita K, Mizuta K, Aoki M (2015). Perfusion imaging of parotid gland tumours: usefulness of arterial spin labeling for differentiating Warthin's tumours. Eur Radiol.

[29] Fujima N, Kameda H, Tsukahara A, Yoshida D, Sakashita T, Homma A (2015). Diagnostic value of tumor blood flow and its histogram analysis obtained with pCASL to differentiate sinonasal malignant lymphoma from squamous cell carcinoma. Eur J Radiol.

[30] Schraml C, Mussig K, Martirosian P, Schwenzer NF, Claussen CD, Haring HU (2009). Autoimmune thyroid disease: arterial spin-labeling perfusion MR imaging. Radiology.

[31] Fujima N, Kudo K, Yoshida D, Homma A, Sakashita T, Tsukahara A (2014). Arterial spin labeling to determine tumor viability in head and neck cancer before and after treatment. J Magn Reson Imaging.

[32] Lin M, Yu X, Ouyang H, Luo D, Zhou C (2015). Consistency of T2WI-FS/ASL fusion images in delineating the volume of nasopharyngeal carcinoma. Sci Rep.

[33] Sun Z, Hu S, Xue Q, Jin L, Huang J, Dou W (2021). Can 3D pseudo-continuous arterial spin labeling perfusion imaging be applied to predict early response to chemoradiotherapy in patients with advanced nasopharyngeal carcinoma?. Radiother Oncol.

[34] Liu Y, Bellomi M, Gatti G, Ping X (2007). Accuracy of computed tomography perfusion in assessing metastatic involvement of enlarged axillary lymph nodes in patients with breast cancer. Breast Cancer Res.

[35] Trojanowska A, Trojanowski P, Bisdas S, Staskiewicz G, Drop A, Klatka J (2012). Squamous cell cancer of hypopharynx and larynx - evaluation of metastatic nodal disease based on computed tomography perfusion studies. Eur J Radiol.

[36] Zhong J, Lu Z, Xu L, Dong L, Qiao H, Hua R (2014). The diagnostic value of cervical lymph node metastasis in head and neck squamous carcinoma by using diffusion-weighted magnetic resonance imaging and computed tomography perfusion. Biomed Res Int.

[37] Noij DP, Martens RM, Marcus JT, de Bree R, Leemans CR, Castelijns JA (2017). Intravoxel incoherent motion magnetic resonance imaging in head and neck cancer: A systematic review of the diagnostic and prognostic value. Oral Oncol.

[38] Driessen JP, van Kempen PM, van der Heijden GJ, Philippens ME, Pameijer FA, Stegeman I (2015). Diffusion-weighted imaging in head and neck squamous cell carcinomas: a systematic review. Head Neck.

[39] Zhang Y, Chen J, Shen J, Zhong J, Ye R, Liang B (2013). Apparent diffusion coefficient values of necrotic and solid portion of lymph nodes: differential diagnostic value in cervical lymphadenopathy. Clin Radiol.

[40] So TY, Ai QH, Lam WKJ, Qamar S, Poon DMC, Hui EP (2020). Intravoxel incoherent motion diffusion-weighted imaging for discrimination of benign and malignant retropharyngeal nodes. Neuroradiology.

[41] Jin GQ, Yang J, Liu LD, Su DK, Wang DP, Zhao SF (2016). The diagnostic value of 1.5-T diffusion-weighted MR imaging in detecting 5 to 10 mm metastatic cervical lymph nodes of nasopharyngeal carcinoma. Medicine (Baltimore).

[42] Lim HK, Lee JH, Baek HJ, Kim N, Lee H, Park JW (2014). Is diffusion-weighted MRI useful for differentiation of small non-necrotic cervical lymph nodes in patients with head and neck malignancies?. Korean J Radiol.

[43] Lai V, Lee VHF, Lam KO, Huang B, Chan Q, Khong PL (2017). Intravoxel incoherent motion MR imaging in nasopharyngeal carcinoma: comparison and correlation with dynamic contrast enhanced MR imaging. Oncotarget.

[44] Yu XP, Hou J, Li FP, Wang H, Hu PS, Bi F (2016). Intravoxel Incoherent Motion Diffusion Weighted Magnetic Resonance Imaging for Differentiation Between Nasopharyngeal Carcinoma and Lymphoma at the Primary Site. J Comput Assist Tomogr.

[45] Chen WB, Zhang B, Liang L, Dong YH, Cai GH, Liang CH (2017). To predict the radiosensitivity of nasopharyngeal carcinoma using intravoxel incoherent motion MRI at 3.0 T. Oncotarget.

[46] Hou J, Yu X, Hu Y, Li F, Xiang W, Wang L (2016). Value of intravoxel incoherent motion and dynamic contrast-enhanced MRI for predicting the early and short-term responses to chemoradiotherapy in nasopharyngeal carcinoma. Medicine (Baltimore).

[47] Lu L, Li Y, Li W (2016). The Role of Intravoxel Incoherent Motion MRI in Predicting Early Treatment Response to Chemoradiation for Metastatic Lymph Nodes in Nasopharyngeal Carcinoma. Adv Ther.

[48] Xiao-ping Y, Jing H, Fei-ping L, Yin H, Qiang L, Lanlan W (2016). Intravoxel incoherent motion MRI for predicting early response to induction chemotherapy and chemoradiotherapy in patients with nasopharyngeal carcinoma. J Magn Reson Imaging.

[49] Liang L, Luo X, Lian Z, Chen W, Zhang B, Dong Y (2017). Lymph node metastasis in head and neck squamous carcinoma: Efficacy of intravoxel incoherent motion magnetic resonance imaging for the differential diagnosis. Eur J Radiol.

