# Supplementary material for: Arterial spin labeling and diffusion-weighted imaging for identification of retropharyngeal lymph nodes in patients with nasopharyngeal carcinoma
Source: Cancer Imaging. 2022 Aug 17;22:40. doi: 10.1186/s40644-022-00480-4 (PMC9387018; doi:10.1186/s40644-022-00480-4)
Supplement: Supplementary file 1 — Additional file 1: Table S1. RLNs size in the three groups. Table S2. Inter-observer consistency and comparisons of the parameters. [file 40644_2022_480_MOESM1_ESM.docx]

**Table1 RLNs size and comparison among three groups**

|  |  | Before treatment | | 1-3 months after radiotherapy | | regression rate (%) | |
| --- | --- | --- | --- | --- | --- | --- | --- |
|  |  | Short axis  (range) (mm) | Long axis (range) (mm) | Long axis (range)  (mm) | Short axis  (range) (mm) | Long axis  (range) | Short axis  (range) |
| LNM (n=71) | | 7.76±4.10  (3-18) | 11.13±5.12  (4-27) | 5.90±2.55  (2-17) | 3.41±1.79 (1-11) | 44.45±13.57  (10.00-78.26) | 52.59±15.67  (0.00-83.33) |
| Non-LNM (n=62) | | 3.60±0.83  (3-7) | 6.17±1.31  (4-11) |  |  |  |  |
| From NPC (n=29) | | 3.45±0.57  (3-5) | 5.93±0.96  (5-9) | 5.72±0.85  (4-8) | 3.25±0.44  (3-4) | 3.75±6.96 (0.00-20.00) | 4.14±9.26  (0.00-25.00) |
| From HC (n=33) | | 3.74±0.98  (3-7) | 6.38±1.54  (4-11) | - | - | - | - |
| Three groups comparison | | **P˂0.001**  (H=71.365) | **P˂0.001**  (H=59.702) |  |  |  |  |
| LNM VS. Non-LNM1^a^ | | **˂0.001** | **˂0.001** |  | - | - | - |
| LNM VS. Non-LNM2^b^ | | **˂0.001** | **˂0.001** |  | - | - | - |
| Non-LNM1 VS. Non-LNM2 | | 1.000 | 1.000 |  | - | - | - |

RLNs = retropharyngeal lymph nodes, S/L = the ratio of short axis to long axis, NPC = nasopharyngeal carcinoma, HC = healthy control.

^a^ Non-LNM1 represents the non-metastatic RLNs from NPC patients.

^b^ Non-LNM2 represents the non-metastatic RLNs from HC participants.

**Table2 Inter-observer consistency and comparisons of the parameters**

|  | BF  (mL/min/100g) | ADC  (×10^-3^mm^2^/s) | D  (×10^-3^mm^2^/s) | D^*^  (×10^-3^mm^2^/s) | f |
| --- | --- | --- | --- | --- | --- |
| ICC (95% confidence interval) | 0.979  (0.970-0.985) | 0.908  (0.870-0.934) | 0.959  (0.943-0.971) | 0.920  (0.887-0.943) | 0.885  (0.838-0.918) |
| LNM (n=71) | 70.33±17.20 | 0.85±0.16 | 0.63±0.12 | 22.11±17.23 | 0.29±0.13 |
| Non-LNM (n=62) | 41.58±13.94 | 1.07±0.32 | 0.74±0.27 | 26.67±22.85 | 0.31±0.13 |
| Patients (n=29) | 43.25±13.05 | 1.10±0.35 | 0.74±0.31 | 28.49±22.86 | 0.32±0.13 |
| Healthy volunteers (n=33) | 40.11±14.73 | 1.04±0.29 | 0.74±0.23 | 25.07±23.07 | 0.31±0.13 |
| Three groups comparison | **P˂0.001**  (H=66.641) | **P****˂0.001**  (H=17.488) | P=0.114 | P=0.557 | P=0.302 |
| LNM VS. Non-LNM1^a^ | **P˂0.001** | **P=0.001** | -^c^ | - | - |
| LNM VS. Non-LNM2^b^ | **P˂0.001** | **P=0.004** | - | - | - |
| Non-LNM1^a^ VS. Non-LNM2^b^ | P=1.000 | P=1.000 | - | - | - |

ICC is the abbreviation of Intra-class correlation.

All the values are calculated as the average of the measurements by two observers.

^a^ Non-LNM1 represents the non-metastatic RLNs from patients.

^b^ Non-LNM2 represents the non-metastatic RLNs from healthy volunteers.

^c^ Multiple comparison were not performed on D, D* and f, because the overall test does not show significant differences across samples.
